# Evaluating the Effect of 3′-UTR Variants in *DICER1* and *DROSHA* on Their Tissue-Specific Expression by miRNA Target Prediction

**DOI:** 10.3390/cimb43020044

**Published:** 2021-07-06

**Authors:** Dmitrii S. Bug, Artem V. Tishkov, Ivan S. Moiseev, Natalia V. Petukhova

**Affiliations:** 1 Bioinformatics Research Center, Pavlov First Saint Petersburg Medical State University, 197022 St. Petersburg, Russia; bug.1@osu.edu (D.S.B.); tishkovav@1spbgmu.ru (A.V.T.); 2 R.M. Gorbacheva Scientific Research Institute of Pediatric Hematology and Transplantation, Pavlov First Saint Petersburg State Medical University, 197022 St. Petersburg, Russia; moisiv@mail.ru

**Keywords:** 3′-UTR variant, miRNA, gene regulation, tissue-specific profiling, *DICER1*, *DROSHA*, myelodysplastic syndrome

## Abstract

Untranslated gene regions (UTRs) play an important role in controlling gene expression. 3′-UTRs are primarily targeted by microRNA (miRNA) molecules that form complex gene regulatory networks. Cancer genomes are replete with non-coding mutations, many of which are connected to changes in tumor gene expression that accompany the development of cancer and are associated with resistance to therapy. Therefore, variants that occurred in 3′-UTR under cancer progression should be analysed to predict their phenotypic effect on gene expression, e.g., by evaluating their impact on miRNA target sites. Here, we analyze 3′-UTR variants in *DICER1* and *DROSHA* genes in the context of myelodysplastic syndrome (MDS) development. The key features of this analysis include an assessment of both “canonical” and “non-canonical” types of mRNA-miRNA binding and tissue-specific profiling of miRNA interactions with wild-type and mutated genes. As a result, we obtained a list of *DICER1* and *DROSHA* variants likely altering the miRNA sites and, therefore, potentially leading to the observed tissue-specific gene downregulation. All identified variants have low population frequency consistent with their potential association with pathology progression.

## 1. Introduction

Differential gene expression analysis is a widely used approach to understand the biological differences between healthy and disease states in order to investigate altered molecular pathways and to discover new targets for specific treatment. RNA sequencing (RNAseq), whole-exome sequencing (WES), and whole-genome sequencing (WGS) are increasingly applied to identify disease-causing genetic variants in individuals [1]. However, despite the current progress in the application of sequencing technologies, analysis of WES or WGS achieves a diagnosis rate of only up to 50% depending on the disease entity [2]. One of the main challenges with the DNA-based molecular diagnostics approaches is the interpretation of non-coding variants. Many non-coding variants play a regulatory role in controlling RNA abundance, splicing and consequent genes expression [3]. A high prevalence of disease-associated SNPs (more than 90%) was found in non-coding regions of the genome, for example in promoter regions, enhancers, and non-coding RNA genes [4,5,6], indicating their important regulatory role. Mutations in 3′-untranslated regions (UTRs) can disrupt, create, or alter the microRNA (miRNA) binding sites thus affecting the expression level of corresponding genes and thereby deregulating pathways that control cellular functions [7]. Noteworthy, we differentiate the terms “mutation” and “polymorphism” as follows: mutation is an event leading to a disease condition, and polymorphism means a variation in the human genome that is observed in populational databases.

miRNAs are estimated to regulate the translation of up to 60% of protein-coding genes [8]; some protein-coding genes are regulated by a single miRNA, while others are regulated by many miRNAs [9]. As fine regulators of gene expression, miRNAs play important roles in key biological processes, such as cell development, proliferation, differentiation, and cell death [10]. miRNAs are a unique class of small (17–22 nucleotides (nt) in length) non-coding single-stranded RNAs that comprise up to 1% of the human genome [11]. miRNAs play an important regulatory role in gene expression at the posttranscriptional level by facilitating sequence-specific RNA interference. The canonical mechanism of their action is the direct interaction with mRNAs of protein-coding genes, followed by degradation of target mRNA by Argonaute-containing silencing complexes [12]. Pairing with mRNA occurs via complementary binding of miRNA seed region (usually nucleotides 2–8 of the miRNA) with the 3′-UTR or, in rare cases, with the 5′-UTR of the target gene [13]. However, recent studies have revealed roles for miRNA sequences beyond the seed region in specifying target recognition and regulation [14], suggesting more complex mechanisms of protein expression control. The newly discovered mechanisms of non-canonical miRNA regulation reveal a wider set of miRNA functions and additional pathways of gene regulation [15].

There are several types of miRNA-mRNA interaction, each leading to a substantial transcriptional repression. Canonical recognition is defined by a perfect pairing of a 6–8-mer subsequence located at the first 7–8 bases of miRNA (seed) and a segment of target mRNA 3′-UTR [12]. The majority of other non-canonical sites do not lead to significant gene downregulation [16,17], however, some specific interactions can be considered as effective as canonical. For example, the 3′-compensatory site, which modulates extensive pairing to the miRNA 3′-region compensates for a wobble or mismatch to one of the seed positions [12,18,19]. Another type of effective non-canonical site is a centered site which represents 11-12 contiguous pairing to the center of the miRNA [20]. Taking into account the complexity of miRNA-mRNA interactions, the alteration of a 3′-UTR can have numerous functional consequences by either introducing or removing miRNA target sequences or changing the binding efficiency, thereby, directly affecting protein expression. Evaluation of such regulatory variants in 3′-UTRs and their potential impact on tissue-specific gene regulation should be performed for individual pathologies that are characterized by aberrant gene expression. For instance, 3′-UTR mutations of *DICER1*, *DROSHA,* and other miRNA processing genes were found to be associated with the increased colorectal cancer risk [21], implying the catastrophic consequences of such, often overlooked and underestimated variants. Moreover, impairment of miRNA-mRNA interaction in miRNA processing genes could lead to altered miRNA expression, which in turn affects the miRNA-mRNA interaction even more, leading to a self-inducing disease mechanism.

Myelodysplastic syndrome (MDS) refers to a heterogeneous group of closely related clonal hematopoietic disorders commonly found in the aging population. All of these disorders are associated with ineffective hematopoiesis with one or more peripheral blood cytopenias and characterized by accumulation of somatic mutations [22] along with genome instability and high incidence of secondary cancerogenic genetic events determining frequent transformation of MDS into acute myeloid leukemia (AML) [23]. The set of genetic alterations defines the disease prognosis and the choice of specific therapy. One of the hypotheses on MDS etiology states that mutations in mesenchymal stromal cells (MSCs) can disrupt the microenvironment which triggers MDS initialization. In particular, deletion of the miRNA processing endonuclease *DICER1* selectively in mesenchymal osteoprogenitors in murine model induces markedly disordered hematopoiesis with several features of MDS, indicating the role of this gene in mesenchymal “stroma” as a primary regulator of tissue function [24]. Moreover, further investigations demonstrated the downregulated expression of both miRNA processing endonucleases DICER1 and DROSHA in MSCs from myelodysplastic syndrome patient cells compared to normal cells. This observation was accompanied by underlying miRNA repression compared to healthy controls [25]. Recent analysis of MDS clinical data revealed a high mutational burden in *DICER1* (54%) and *DROSHA* (17%), and the detected SNPs were predominantly located in 5′-UTR and 3′-UTR regions [26]. Taken together, these observations suggest that during MDS progression, *DICER1* and *DROSHA* genes are under mutational pressure directed at the dysregulation of miRNA processing function and, as a result, leading to impaired protein expression. The triggering mechanism of such global dysregulation might be due to increased targeting of miRNA or by newly formed miRNA pairing to *DICER1* and *DROSHA* due to the acquired mutations in regulatory regions (mainly in 3′-UTR) during tumor progression. Consequently, the present study is focused on evaluation of 3′-UTR SNPs in *DICER1* and *DROSHA* genes through the prediction of putative miRNA target subsequences and their comparison due to the acquired mutations. SNP prioritization is based on bioinformatics analysis of structural and functional properties of miRNA pairing to the altered and original 3′-UTR loci. We propose to filter out those miRNAs that are not detectable in the normal tissue; here, we used the miRNA profile of MSCs that is specific to the scope of MDS. The proposed pipeline can be further applied to evaluate noncoding SNPs in other genes and tissues of interest.

## 2. Materials and Methods

### 2.1. Data Collection

All known 3′-UTRs polymorphisms of *DICER1* and *DROSHA* were taken from dbSNP (https://www.ncbi.nlm.nih.gov/snp/, accessed on 5 February 2021), as well as mutations discovered in probes with “haematopoietic and lymphoid” histology subtype from COSMIC database (https://cancer.sanger.ac.uk/cosmic, accessed on 5 February 2021) and from the recent study of these genes in patients with MDS [26]. 3′-UTR sequences were taken from NM_001195573 transcript for *DICER1*, and from NM_013235 transcript for *DROSHA*.

### 2.2. ”Canonical” Binding Analysis

MicroSNiPer (MPI-IE, Freiburg, Germany) [27] tool interrogates the 3′-UTR and predicts whether a SNP within the target site will disrupt/eliminate or enhance/create a miRNA binding site. It applies the modified FASTA algorithm [28] for finding significant matches between 3′-UTR and miRNAs from miRbase database release v19 (http://www.mirbase.org/, accessed on 17 February 2021) [29]. The alignment between the 3′-UTR and the miRNA requires an uninterrupted match of at least 6 nucleotides from the 5′-end of miRNA which would mimic the canonical seed site. In our analysis, we used the following parameters: a minimal length set to 6 nucleotides and G-U wobbles allowed. A table of miRNAs was derived with their corresponding seeds at the original and mutated 3′-UTR of *DROSHA* and *DICER1*.

### 2.3. Energy Evaluation of mRNA-miRNA Duplex

The IntaRNA v2.0 (University of Freiburg, Freiburg, Germany) [30] algorithm implements the RNA energy parameters from the Vienna RNA package v2.* (University of Vienna, Vienna, Austria) [31], enforcing the seed interaction to be energetically favorable. It was used with default parameters (single-site, loop-based RNA-RNA interaction with minimal free energy model, taking only energetically favorable interactions with negative energy, the temperature is set to 37 °C) on the set of original and mutated 3′-UTR subsequences of 50 nucleotides against the miRNAs of the whole miRbase database release v19 [29]. This tool was also used to supply MicroSNiPer results with their duplex energy values: to be consistent, each miRNA predicted by MicroSNiPer was tested to form exactly the same hybrid configuration in IntaRNA. The total number of predicted miRNA-RNA interactions was 74,977 (73,178 from IntaRNA and 1799 from MicroSNiPer). Sites with insufficient binding energy were omitted with the limit of −8.5 kcal/mol [32].

### 2.4. Tissue-Specific miRNA Profiling

The miRNA profile in MSCs was taken from Clark et al., 2014 [33]. From this set, 26 miRNAs derived from human bone marrow MSCs were used as the most abundant miRNAs in MSCs. In a more recent study, miRNAs of bone marrow-derived MSCs were also studied [34,35]. Moreover, it was shown that MSCs and MSCs’ exosomal vesicles have, in general, similar miRNA expression [36]. We built the final set of 336 miRNAs from all available publications dealing with the bone marrow-derived MSCs exosomes [35,37,38,39,40]. 

### 2.5. Resulting Genes Variant Selection

miRNAs that were shown to be abundant in MSCs were used in the main datasets. Additionally, sets of miRNAs commonly detected in MSCs were used as an additional dataset. Two main parameters were calculated for each variant: the change in the number of miRNAs hybridizing to the 3′-UTR, and the difference in the sum of hybridization energies. Distributions of both parameters were analyzed, and the “statistical outliers” were taken further as the most significant result. The variants that lead to an increase in the number of miRNAs and a decrease in hybridization energy sum are shown in Table 1 and Table 2. All analyzed variants, the data of interacting miRNAs and the code can be found at the links provided in the Data Availability Statement. Population allele frequencies were obtained for selected variants from dbSNP, which collects data from multiple databases (ALFA (NCBI, Bethesda MD, USA), 1000Genomes (EMBL-EBI, Hinxton, Cambridgeshire, UK), GnomAD (Broad Institute, Cambridge, MA, USA), TOPMED (University of Washington, Seattle, WA, USA), ALSPAC (University of Bristol, Bristol, UK), TWINSUK (King’s College London, London, UK), Genetic variation in the Estonian population (University of Tartu, Tartu, Estonia), Northern Sweden (Uppsala University, Uppsala, Sweden), GoNL (The Netherlands), KRGDB (National Institute of Health, Cheongju, Korea), and A Vietnamese Genetic Variation Database (Vietnam National University, Hanoi, Vietnam)).

## 3. Results

### 3.1. Building the Pipeline for 3′-UTR SNPs Analysis Considering Tissue-Specific miRNA Expression

General approach to evaluate the effect of 3′-UTR SNPs on miRNA binding is based on prediction of the miRNA-mRNA interaction. Most of the current tools mainly compute the putative binding sites by estimating the strict seed pairing along with site accessibility, conservation, and base pairing stability [41,42,43,44,45,46]. Some methods specialize on prediction of the SNP impact on putative miRNA targets [27,47,48]. In order to build the generalized approach for miRNA target prediction depending on the acquired SNPs, the pipeline should cover the following important features: (1) evaluate both “canonical” sites and “non-canonical” miRNA binding with 3′-UTR, known as effective expression regulators [49], on the basis of the defined set of interaction patterns and hybridization energies [50,51]; (2) analyze not only catalogued variants but also novel SNPs and indels; (3) allow tissue-specific prediction of found miRNAs for altered gene sites, because the miRNA composition varies across tissues [52].

**Figure 1:**
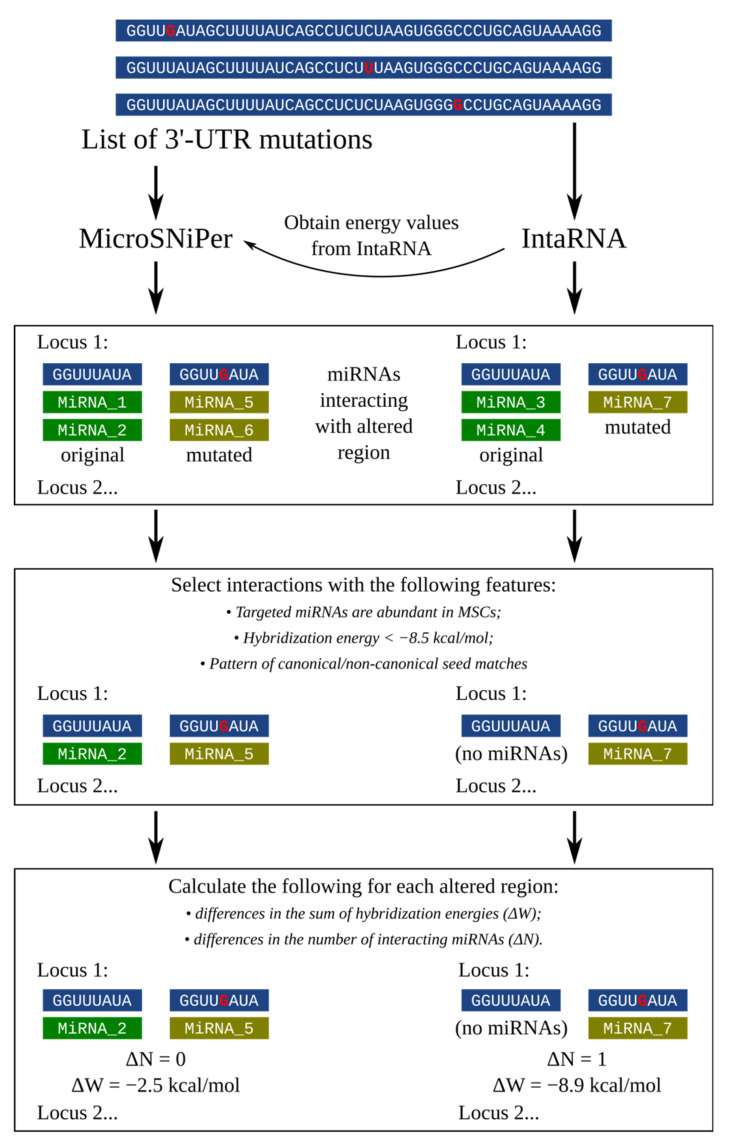
The general workflow for prediction of 3′-UTR mutations effect on miRNA tissue-specific targeting. The pipeline shows the process of evaluating miRNA targets for altered and original *DICER1* and *DROSHA* loci in the context of MDS (tissues-specific analysis in MSCs). Mutation sites are indicated in red.

Taking these features into account, we propose the pipeline shown in Figure 1. The MicroSNiPer application has been chosen as the only one flexible, customizable tool to analyze any input SNP position in 3′-UTR sequence. This tool interrogates the 3′-UTR and predicts whether a SNP within the target site will disrupt/eliminate or enhance/create a miRNA binding site. MicroSNiPer’s algorithm is based on seed region binding in the miRNA as the major criterion for prediction [27]. The important feature of MicroSNiPer is the characterization of both novel SNPs and “in-house” transcripts. In addition to the “seed-oriented” method of “canonical” miRNA target evaluation, the IntaRNA algorithm was incorporated into the proposed pipeline. It represents one of the most widely used state-of-the-art approaches for general RNA–RNA interaction prediction. It enables screening a large target sequence and searching the most energetically favorable interactions, incorporating seed constraints and interaction site accessibility along with enhanced parameterization as well as fully customizable control [30]. The main feature introduced in the proposed pipeline is filtering the variants with the most altering potential for tissue-specific miRNA binding compared with the original sequence. The cutoff value for hybridization energy can be chosen for each RNA-RNA duplex followed by selection of only those interactions with miRNAs that are abundant in specific tissue of interest.

**Figure 2:**
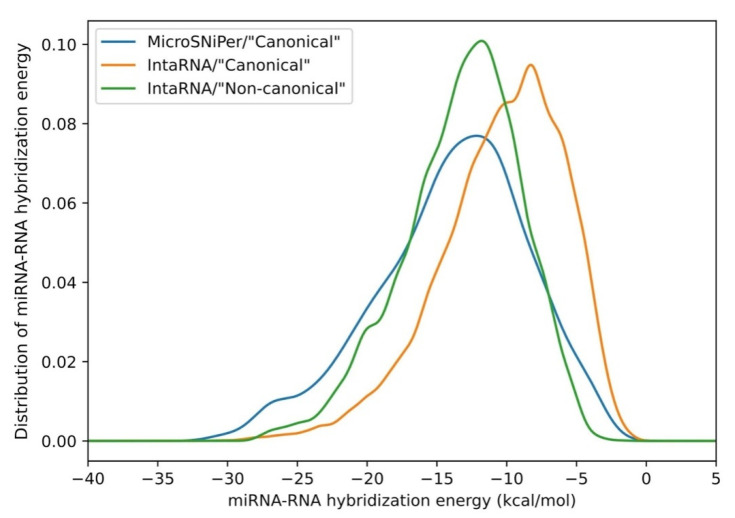
The distribution of miRNA-RNA hybridization energy values for all predicted interactions between miRNA and *DROSHA* 3′-UTR with and without SNPs. MicroSNiPer finds mostly strong canonical seeds compared to IntaRNA. The difference between “canonical” and “non-canonical” interaction energies calculated by IntaRNA is probably due to the higher demand of “non-canonical” seeds for internucleotide bonds.

Hereby, the lists of interacting miRNAs were generated for each target allele (original and mutated) of each locus (a subsequence in the vicinity of which the variant occured) for *DICER1* and *DROSHA* by using MicroSNiPer and IntaRNA algorithms. The distribution plot of hybridization energies for all predicted interactions between miRNA and *DROSHA* 3′-UTR for original and mutated loci (Figure 2) demonstrates slight differences in results obtained by the two methods. Our pipeline covers evaluation of both “canonical” and “non-canonical” types of interactions, considering their thermodynamic significance for miRNA-mRNA hybridization.

Only a subset of miRNAs that are found in MSCs was taken into consideration. Interaction sites with insufficient (> −8.5 kcal/mol) hybridization energy were discarded as this energy value was shown to be required for suboptimal duplex formation [32]. Moreover, all interactions were validated to match a pattern of canonical (6–8-mer site covering the first 7–8 bases of miRNA) or non-canonical seeds (3′-compensatory site and centered site, described earlier).

Finally, for each locus, differences in sums of hybridization energies with all interacting miRNAs were calculated, as well as the differences in numbers of interacting miRNAs between original and mutated allele. The “statistical outliers” as the most significant distinctive values identified the variants that probably shift the gene expression. *DICER1* and *DROSHA* were shown to be downregulated in patients with MDS [25], therefore, we focused only on variants that increase the target mRNA affinity for the pool of miRNAs, therefore, decreasing the overall gene expression.

### 3.2. Variants in DROSHA 3′-UTR Predicted to Downregulate Gene Expression

Using our pipeline, we analyzed 211 3′-UTR variants of *DROSHA* (193 SNPs and 18 indels). Variants that are predicted to downregulate gene expression (showing significant difference in miRNA binding compared to the original sequence) are listed in Table 1. It can be concluded the majority of efficient downregulating variants were predicted by MicroSNiPer having “canonical” seed interactions with found miRNA in MSCs. Nevertheless, the results of both algorithms should be considered side-by-side and complement each other as they are based on different sets of duplexes formed.

### 3.3. Variants in DICER1 3′-UTR Predicted to Downregulate Gene Expression

The total 959 *DICER1* 3′-UTR variants (854 SNPs and 105 indels) were analyzed with respect to their impact on the gene expression. Variants with the highest potential to downregulate gene expression are listed in Table 2. These 64 SNPs are likely to influence the *DICER1* expression in MSCs. *DICER1* has an unusually long 3′-UTR (>4000 nt) [53], which implies high potential for miRNA binding and might indicate the importance of post-transcriptional gene expression regulation. Moreover, it was shown to be targeted by several miRNAs, for example, miR-103/107, miR-192 [54] and miRNAs of let-7 family [55]. Therefore, the variants found to be altering the miRNA binding in MSCs, especially under mutational pressure in cancer progression, might have an impact on gene expression.

## 4. Discussion

Advances in high-throughput sequencing technologies have revolutionized the field of medical genetics, providing important insights into the genetic basis of many diseases, and has opened the path to promising preventive, diagnostic, and therapeutic strategies [56]. It is widely accepted that MDS-related mutations occur mainly in genes involved in mRNA splicing, epigenetic regulation, signal transduction, transcription regulation, tumor suppression, and adhesion [57,58]. Recent studies have demonstrated that mutations in miRNA-processing endonucleases DICER1 and DROSHA are associated with MDS progression [26,59]. Impaired *DICER1* and *DROSHA* expression was reported in mesenchymal stromal cells of MDS patients [25]. Taken together the observed downregulation of these genes in some MDS patients [25] and significant prevalence of mutations in their UTRs [26], call for analysis of these variants in the context of *DICER1* and *DROSHA* regulation, specifically during MDS progression.

miRNAs are potential biomarkers [60] for diverse pathologies including oncology [61,62,63], cardiovascular diseases [64,65], and sepsis [66]. The significance of these regulatory molecules has arisen from distinct and complex interaction networks of miRNA and their target mRNA, which defines the expression profile in the cell [67,68,69].

Due to the important role of miRNAs in gene regulation, identification of their targets is essential, especially in terms of mutational pressure in cancer along with dysregulation of protein expression. Although the experimental approaches to evaluate the functional effect of SNPs in 3′-UTR exist [70,71,72], they are highly demanding in terms of resources and time, and the assessment of all known gene mutations at once is not always feasible. Therefore, the accurate in silico methods for predicting functional consequences of SNPs that are found in 3′-UTRs are necessary to guide further experiments. Here, we present a modified pipeline for analysis of 3′-UTR variants, which takes into account both “canonical’ and “non-canonical” miRNA target evaluation as well as tissue-specific miRNA expression. Several previous studies aimed to analyze non-coding regulatory variants and to predict their functional effect on disease progression. For example, Sabina et al., 2015 [50] calculated the minimum free energies by applying each miRNA to the 3′-UTR sequence of the *H2AFX* gene using the RNAcofold tool from Vienna RNA suite [31]. When the whole 3′-UTR sequence is used in RNAcofold, complementary sites may emerge, establishing intra-molecular loops that can shield the potential binding site. Other oligo- and polynucleotides, as well as the rest of the target RNA sequence may also interfere with potential miRNA-binding sites. Since effects of many possible interactions in the complex cell environment cannot be accurately predicted, we proposed to use two sets of short (50 nt) subsequences of the 3′-UTR sequences in the variant vicinity as a target in our pipeline. The search of expression-changing candidate miRNAs should be based on two main criteria: (1) calculation of thermodynamic properties (implemented in, for instance, the Vienna RNA package v2.* [31], which is incorporated in the IntaRNA [30] algorithm), and (2) classification of targets as effective and ineffective, which may come from definitions of “canonical” and “non-canonical” seeds or from applying deep learning methods [73]. That is why the algorithms of MicroSNiPer (a “canonical” interaction predictor) and IntaRNA (an extended flexible RNA-RNA binding analyzer) were incorporated into the proposed pipeline in order to evaluate a wide spectrum of miRNA targeting sites. Moreover, it should be noted that each tissue possesses its own miRNA profile, and consequently the set of query miRNAs should be limited to miRNAs that are abundant (or at least shown to exist) in the cells of a studied tissue. The present pipeline can be applied for evaluation of other genes targets in various tissues of interest.

In conclusion, we analyzed a set of 3′-UTR variants in *DICER1* and *DROSHA* genes, and, as a result, identified mutations with the highest potential impact on the miRNA binding in MSCs. Population analysis showed that all variants predicted to downregulate *DICER1* and *DROSHA* can be considered as rare (population allele frequency < 0.5%). This resulting list of candidate variants should be studied experimentally to reveal their association with downregulation of gene expression and involvement in MDS progression.

## Figures and Tables

**Table 1:** The most effective variants predicted to downregulate the *DROSHA* expression in mesenchymal stromal cells (MSCs).

Method	Variant Name	Change in the Number of Interacting miRNAs (ΔN)	Change in the Sum of Hybridization Energies (ΔW, kcal/mol)	Maximum Population Allele Frequency
IntaRNA	rs1479981622:C>T	5	−62.01	8 × 10^−6^
	rs536092006:T>C	5	−61.06	0.0003
	rs1049043869:C>G	6	−55.91	6.4 × 10^−5^
MicroSNiPer	rs775376244:T>C	2	−24.60	0.0005
	rs550965893:T>G	2	−22.41	0.0002
	rs771705336:C>G	1	−17.85	0.0003
	rs766853644:T>C	1	−17.68	8 × 10^−6^
	rs1362918185:G>A	1	−16.64	2.4 × 10^−5^
	rs536221210:A>G	1	−16.57	Not provided
	rs1221977896:A>C	1	−16.30	2.4 × 10^−5^
	rs752035593:G>A	1	−15.81	3.2 × 10^−5^
	rs1260437486:G>A	1	−15.56	8 × 10^−6^
	rs1014491371:C>G	1	−15.29	8 × 10^−6^
	rs761935330:G>A	1	−15.03	0.0003
	rs1441949563:C>T	1	−14.07	3 × 10^−5^
	rs1033831185:A>G	1	−11.70	3 × 10^−5^
	rs1358226278:C>G	1	−11.38	8 × 10^−6^
	rs1424327104:C>T	1	−11.11	Not provided
	rs1288407577:T>G	1	−10.58	4 × 10^−6^
	rs1187772510:A>G	1	−8.98	3.2 × 10^−5^

**Table 2:** The most effective variants predicted to downregulate the *DICER1* expression in mesenchymal stromal cells (MSCs).

Method	Variant Name	Change in the Number of Interacting miRNAs (ΔN)	Change in the Sum of Hybridization Energies (ΔW, kcal/mol)	Maximum Population Allele Frequency
IntaRNA	rs1296755923:T>G	9	−126.61	0.0003
	rs1207839989:A>G	8	−101.32	3 × 10^−5^
	rs1041875974:G>T	6	−98.42	Not provided
	rs981079616:T>G	6	−94.19	8 × 10^−6^
	rs760246677:G>T	6	−91.69	8 × 10^−6^
	rs989262025:A>G	6	−84.46	3.3 × 10^−5^
	rs1281573015:A>T	8	−83.06	8 × 10^−6^
	rs1248738927:G>C	6	−82.47	Not provided
	rs1221590835:G>A	7	−79.94	3 × 10^−5^
	rs1324216335:T>C	7	−74.46	8 × 10^−6^
	rs1206166531:C>G	5	−71.61	0
	rs1194042023:A>G	5	−68.88	3.2 × 10^−5^
	rs889120755:C>G	5	−68.86	Not provided
	rs571735282:A>G	5	−65.47	0.0012
	COSN31961029:T>C	5	−65.08	Not provided
	rs902240610:A>G	5	−64.83	8 × 10^−5^
	rs552609115:T>C	5	−64.25	Not provided
	rs930259829:T>G	7	−63.86	4.8 × 10^−5^
	rs1206166531:C>T	5	−58.35	0.0002
	rs535308545:A>C	5	−58.35	3.2 × 10^−5^
	rs1381450764:G>A	6	−57.20	Not provided
	rs923348310:C>T	5	−53.65	7.2 × 10^−5^
	rs752666806:C>T	5	−45.40	Not provided
MicroSNiPer	rs565097712:G>A	7	−77.40	0.0004
	rs1396697573:A>G	2	−41.98	Not provided
	rs1307365736:A>T	2	−30.12	8 × 10^−6^
	rs895962647:A>T	2	−27.72	3.2 × 10^−5^
	rs942463789:T>G	1	−24.94	0.0003
	rs997751105:C>T	2	−22.40	1.6 × 10^−5^
	rs926249853:T>G	2	−20.43	8 × 10^−6^
	rs1478240035:G>T	1	−19.62	8 × 10^−6^
	rs1405544334:G>T	1	−19.59	8 × 10^−6^
	rs1273786688:T>G	2	−18.42	0.005
	rs1045581145:A>T	1	−17.48	8 × 10^−6^
	rs889120755:C>G	1	−16.79	Not provided
	rs923753708:G>C	1	−16.70	Not provided
	rs1461667204:T>A	1	−16.60	8 × 10^−6^
	rs528282193:C>T	1	−16.60	0.001
	rs1338875884:A>G	1	−16.32	3 × 10^−5^
	rs1165916168:C>T	1	−16.04	Not provided
	MU75396710:C>G	1	−15.66	Not provided
	rs1394767633:A>G	1	−15.34	3.2 × 10^−5^
	rs1288975386:T>A	1	−14.99	Not provided
	rs752666806:C>T	1	−14.39	Not provided
	rs1253019517:T>A	1	−14.31	1.6 × 10^−5^
	rs1219762419:A>G	1	−13.75	6 × 10^−5^
	rs1371551409:A>C	1	−13.69	8 × 10^−6^
	rs1181773370:C>T	1	−12.71	3 × 10^−5^
	rs1452738847:A>G	1	−12.03	8 × 10^−6^
	rs1467042427:A>G	1	−11.51	8 × 10^−6^
	COSN25075844:T>C	1	−11.48	Not provided
	COSN25075844:T>C	1	−11.48	Not provided
	MU85472748:T>C	1	−11.48	Not provided
	MU85472748:T>C	1	−11.48	Not provided
	rs972230345:A>G	1	−11.44	4 × 10^−5^
	rs887676239:A>G	1	−11.12	5.6 × 10^−5^
	rs959339279:A>G	1	−11.00	0.0001
	rs971064154:A>G	1	−10.68	8 × 10^−6^
	rs1205529398:G>A	1	−9.88	8 × 10^−6^
	rs1306390419:A>G	1	−9.49	8 × 10^−6^
	rs1292049568:G>T	1	−9.33	8 × 10^−6^
	rs982013263:G>T	1	−9.17	6.4 × 10^−5^
	rs914976738:C>T	1	−8.97	Not provided
	rs1488494352:A>G	1	−8.60	8 × 10^−6^

## Data Availability

The data presented in this study are openly available via following links: all analyzed variants are listed in supplementary tables (http://epicenter.1spbgmu.ru/static/files/all_variants.zip, accessed on 6 July 2021); the raw data of interacting miRNAs with corresponding variants and their hybridization energies were deposited at http://epicenter.1spbgmu.ru/static/files/json.zip, accessed on 6 July 2021; the code is available online at http://epicenter.1spbgmu.ru/static/files/DICER1_DROSHA_miRNA_research.zip, accessed on 6 July 2021.
